# Omalizumab for STAT3 Hyper-IgE Syndromes in Adulthood: A Case Report and Literature Review

**DOI:** 10.3389/fmed.2022.835257

**Published:** 2022-05-04

**Authors:** Jun Lan, Yi Zhang, Min Song, Shan Cai, Hong Luo, Ruoyun OuYang, Pan Yang, Xiaoliu Shi, Yingjiao Long, Yan Chen

**Affiliations:** ^1^Department of Medical Genetics, The Second Xiangya Hospital, Central South University, Changsha, China; ^2^Department of Gastroenterology, The Second Xiangya Hospital, Central South University, Changsha, China; ^3^Division of Pulmonary and Critical Care, The Second Xiangya Hospital, Central South University, Changsha, China; ^4^Division of Pulmonary and Critical Care, Hengdong County People's Hospital, Hengyang, China

**Keywords:** hyper-IgE syndrome, STAT3 gene, allergy bronchopulmonary aspergillosis, omalizumab, case report

## Abstract

**Background:**

Hyper-immunoglobulin E (IgE) syndromes (HIES) are a group of primary immune deficiencies disorders (PID) characterized by elevated serum IgE, eczema, recurrent skin, or respiratory system infections and may also be accompanied by some connective tissues and skeletal abnormalities. Currently, there is no complete cure or targeted treatment for HIES. Omalizumab is a humanized recombinant monoclonal antibody against IgE, reducing the level of free IgE, inhibiting the binding of IgE to receptors on the surface of effector cells, and reducing the activation of inflammatory cells and the release of multiple inflammatory mediators. However, the effect of omalizumab in treating HIES remains unknown. Herein, we described a case of an AD-HIES patient with chronic airway disease who benefited from omalizumab treatment.

**Case Presentation:**

A 28-year-old Chinese woman was admitted for recurrent cough for 7 years, markedly elevated serum IgE level, and recurrent pneumonia caused by multiple pathogens, such as *Pneumocystis jirovecii, Cytomegalovirus, Staphylococcus aureus, Aspergillus*, and *Mycobacterium tuberculosis*. She had eczema-dermatitis, skin abscess, slightly traumatic fracture since childhood, and developed asthma and allergic bronchopulmonary aspergillosis (ABPA) lately. Using whole-exome sequencing, the *STAT3* (c.1294G>T, p.Val432Leu) missense mutation for the autosomal dominant hyper-IgE syndrome was identified, and omalizumab was prescribed at 300 mg every 2 weeks. The patient responded well with the improvement of respiratory symptoms and lung function tests. The level of serum IgE remained stable on follow-up.

**Conclusion:**

Omalizumab treatment proved beneficial in the case of HIES, especially with chronic airway disease, for which therapeutic options are limited. However, larger-scale prospective studies and long-term follow-up are required to establish the efficacy and safety of this therapeutic intervention.

## Introduction

Hyper-IgE syndromes (HIES) are a group of primary immunodeficiency disorders (PID) characterized by a triad of eczema, recurrent skin and pulmonary infections, and the elevated serum IgE level (1). According to the different genetic modes, it can be divided into autosomal dominant heredity (AD-HIES, also known as Job syndrome, OMIM 147060) and autosomal recessive heredity (AR-HIES, OMIM 243700). However, existing cases are sporadic. The autosomal dominant form of HIES results from the dominant-negative loss of function mutations of STAT3, a signal transducer and an activator of transcription 3 (2, 3), which is unique in non-immune manifestations, including retained primary teeth, scoliosis, bone fractures following minimal trauma, joint hyperextensibility, and characteristic facial appearance (4). The non-immunological phenotypes are less common in patients with AR-HIES who fulfill the HIES clinical trial (5).

Autosomal dominant heredity-HIES is a rare disease with a morbidity of 1/1,000,000 per year (6). Enrichment in a specific ethnic or racial group has not been reported, and no genotype-phenotype correlations for *STAT3* pathogenic variants have been identified. Therefore, the disease is easy to be misdiagnosed or missed. There is currently no effective treatment available either. Intensive care of skin lesions, prompt antibiotic and antimycotic treatment for infections, and surgical drainage of abscesses are the mainstays of HIES management. Bone marrow transplantation has been implied recently (7, 8). Omalizumab, the humanized recombinant monoclonal antibody against IgE, is known to result in a marked reduction in serum-free IgE and the downregulation of IgE receptors on circulating basophils (9). Some case reports indicated that omalizumab successfully improved skin symptoms and may be associated with decreased serum IgE during therapy (9–14). However, the data remains scarce. Herein, we described the case of a patient with AD-HIES treated with omalizumab for 6 months with optimistic efficacy.

## Case Descriptions

**Figure 1:**
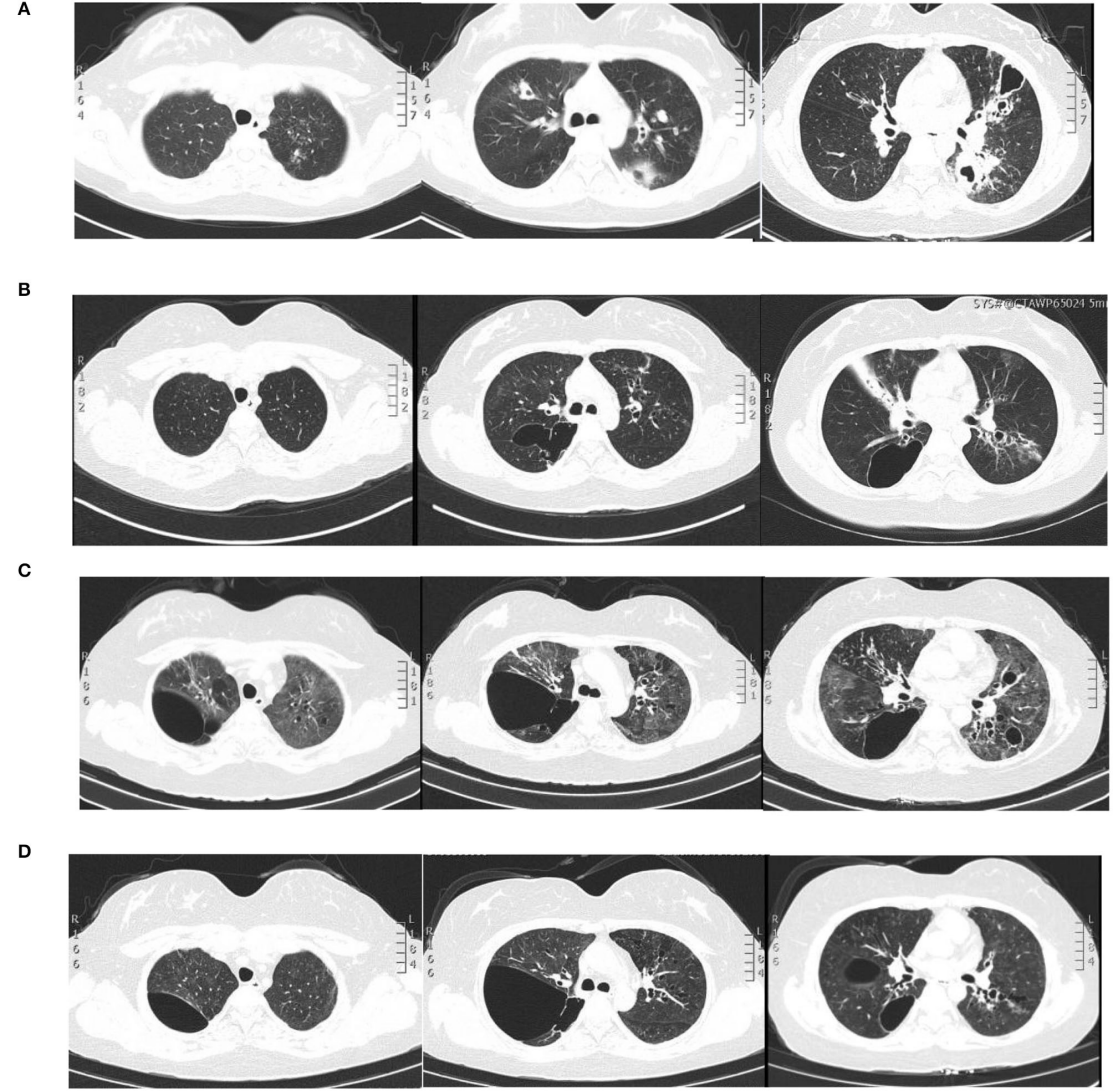
Pulmonary imaging by CT scan. **(A)** December 2015: multiple cavitary lesions and multiple patchy shadows in both the upper lungs and the dorsal segment of the left lower lung, with bronchiectases in the right middle lung and the lingual and dorsal segments of the left upper and left lower lung. **(B)** December 2020: multiple cystic and columnar dilated bronchial shadows of variable size in both lungs, with exudative foci in the middle lobe of the right lung. **(C)** 8 May 2021: diffuse multiple ground glass shadows and scattered striae in both lungs and more cystic lesions than previously observed. **(D)** 18 August 2021: improved infiltrations and multilobed segmental bronchiectases and bullae in both lungs.

A 28-year-old woman was admitted to the Second Xiangya Hospital of Central South University, Hunan, China because of recurrent cough for 6 years and wheezing for 7 months, which aggravated for half a month in May 2021. The patient started to cough sputum early in September 2014, and pulmonary tuberculosis was considered the reason at the local hospital and therefore anti-tuberculosis was given. The symptoms improved significantly until 2015; the patient complained of cough with fever during the anti-tuberculosis therapy and visited our hospital for the first time. Laboratory tests showed an elevated level of leukocyte count, erythrocyte sedimentation rate, C-reactive protein level, and serum total IgE (2,500 IU/ml, normal range: <130 IU/ml). The chest CT revealed multiple cavitary lesions in both upper lungs and the dorsal segment of the left lower lung and bronchiectasis of bilateral lobes (Figure 1A). A combined infection of aspergillosis and *Staphylococcus aureus* was confirmed by positive culture of both sputum and bronchoalveolar lavage fluid (BALF). Anti-infection with teicoplanin and itraconazole was prescribed, and her symptoms were relieved on therapy. The patient remained stable until 2019; she began coughing intermittently and the symptoms gradually worsened when accompanied with dyspnea and wheezing. She visited our hospital for the second time in December 2020. Blood tests showed a moderately elevated level of eosinophil count (0.97 × 10^9^/L, normal range: 0.02–0.52 × 10^9^/L) and a markedly increased serum total IgE level (>2,500 IU/ml). Total levels of aspergillosis-specific IgE, IgM, and IgG were above normal. A pulmonary function test revealed decreased FVC (2.41 L; expected value: 2.92 L), decreased FEV1 (1.54 L, expected value: 2.53 L), and decreased FEV1/FVC (63.68%, expected value: 84.52%), indicating moderate obstructive pulmonary ventilation dysfunction and positive bronchodilation test (FEV1 increased by 16.8%). The chest CT revealed bilateral multiple bronchiectases, pulmonary bulla, and atelectasis in the right middle lung lobe (Figure 1B). Based on these data, a diagnosis of allergic bronchopulmonary aspergillosis (ABPA) was made. Oral voriconazole 200 mg two times daily and methylprednisolone 16 mg per day plus inhaler were prescribed but were demonstrated to be insufficient without the improvement of symptoms. Since February 2021, she was given omalizumab 600 mg three times every month; she was readmitted due to the aggravation of cough and wheezing. The patient denied any history of food or drug allergies. Her family history was negative for any inherited genetic diseases.

**Figure 2:**
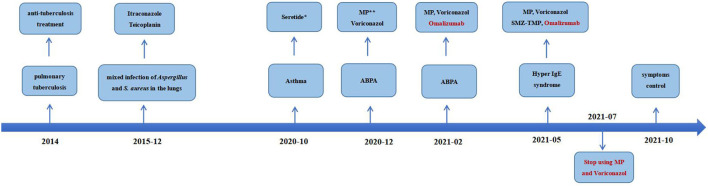
Time-line with the most relevant data of the clinical case.*Salmeterol Xinafoate and Fluticasone propionate powder for inhalation. **Methylprednisolone.

On physical examination, her body weight was 58 kg on admission. She exhibited facial flushing, a full moony face, buffalo back, and mossy skin changes on the scalp. Moist rales could be heard on bilateral lung fields. Her laboratory findings on admission revealed moderate C-reactive protein level (33.6 mg/l, reference range: 0–6 mg/l) and a normal level of leukocyte count and erythrocyte sedimentation. Serum IgE was above normal (770 IU/ml, reference range: <130 IU/ml); serum (1–3)-beta-D-glucan test, galactomannan test, anti-nuclear antibody, anti-neutrophil cytoplasmic antibodies, vasculitis antibodies, antiphospholipid antibodies, antibodies to extractable nuclear antigens, and rheumatoid factor were negative. Pulmonary CT suggested diffuse multiple ground glass shadows and scattered striae in both lungs and more cystic lesions than previously observed (Figure 1C). Bronchoscopy showed viscous jelly-like secretions in the tracheal lumen. The next-generation sequence (NGS) of BALF indicated *Pneumocystis jirovecii* (sequence reads 34909), Human Herpes Virus Type 5 (also named CMV, sequence reads 1843), and Human Herpes Virus Type 4 (also named EBV, sequence reads 7). With classic diffuse ground-glass lesions appearing in the chest CT images and high sequence reads of CMV and *P. jirovecii* in BALF detected by mNGS, we identified that the patient had CMV and *P. jirovecii* coinfection. The sequence reads of EBV, which is ubiquitous in most of the population, were too few to be identified as pathogenic. SMZ-TMP and ganciclovir were given and symptoms were gradually relieved (Figure 2).

**Figure 3:**
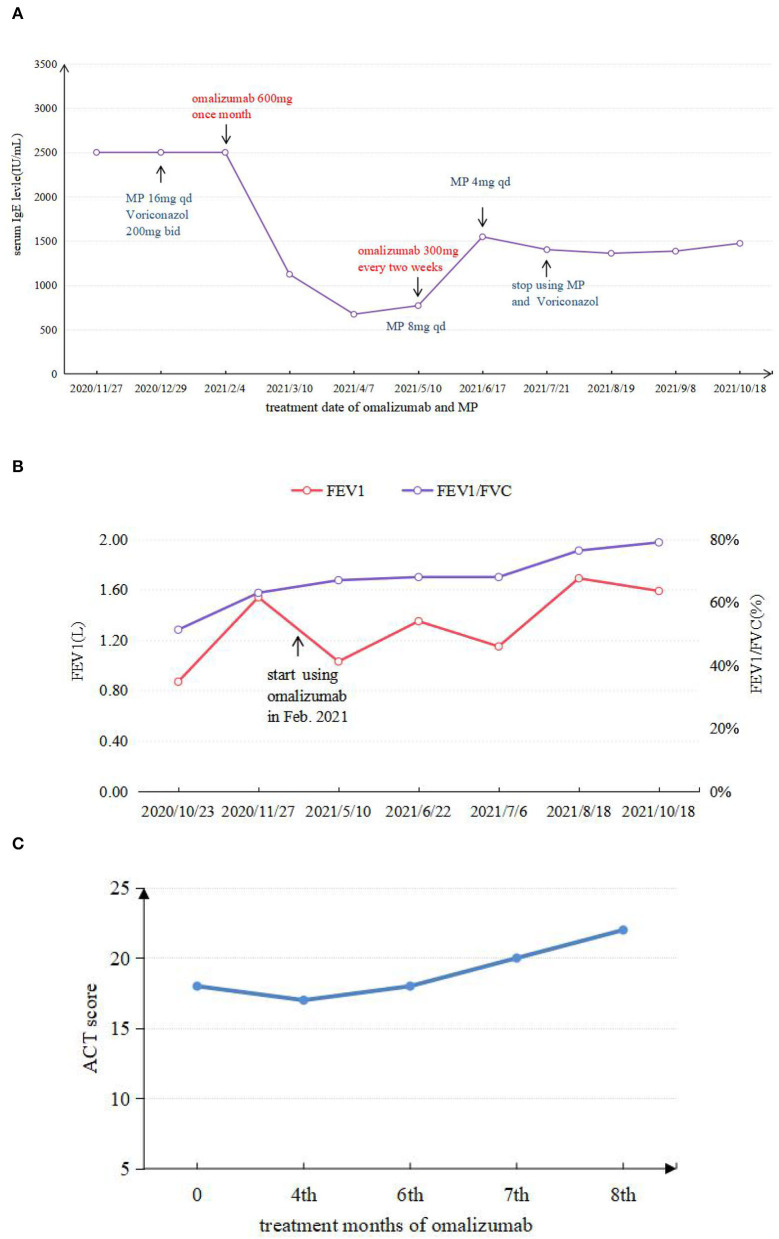
**(A)** Comparison between IgE levels during application of omalizumab and MP. **(B)** FEV1 and FEV1/FVC test results in different periods. **(C)** ACT score results in different periods.

Given that the young patient was predisposed to multiple pathogens, including some opportunistic strains, we carefully reviewed the patient's medical history. Surprisingly, she had multiple skin rashes and pruritus all over the body since childhood. A minor traumatic fracture of the right calf occurred at the age of 12. She underwent cutaneous abscesses requiring incision and drainage at 13 years of age. The patient also had a previous diagnosis of pelvic abscess, tubal occlusion, and primary infertility. The patient had recurrent infections of different strains including opportunistic pathogens in multiple organs, skin rashes, and markedly elevated IgE levels. HIES were suspected, and she scored 45 points on the National Institutes of Health clinical HIES scoring system (15). Further whole-exome sequencing (WES) was performed to screen potential mutations, indicating a heterozygous missense mutation in exon 15 of *STAT3* (c.1294G>T, g.62382G>T, p.Val432Leu, NM_139276.2). Consequently, the final diagnosis was made as AD-HIES. Her parents and younger sister are wild-type genotypes. In addition to the standard anti-infection therapy, we prescribed omalizumab 300 mg every 2 weeks and Seretide inhalation. Oral corticosteroids gradually reduced and stopped. During 6 months of follow-up, the patient did not experience recurrent infection. After an initial fluctuation of the level, serum IgE remained stable recently. The patient's symptoms and lung function improved on therapy (Figure 3). The chest CT scan demonstrated improved infiltrates (Figure 1D).

## Discussion and Conclusion

We herein reported an AD-HIES case with a missense mutation of the STAT3 gene responding well to omalizumab. AD-HIES were first described as Job's syndrome in 1966. In the last decades, advances in genetic testing have allowed the identification of potential genetic causes for HIES. Heterozygous mutations with dominant-negative effects in signal transducer and activator of transcription 3 (STAT3) genes are thought to be responsible for AD-HIES.

The *STAT3* gene is located on chromosome 17q21, contains 24 exons, and spans 30 kb. It encodes a signal transducer and an activator of transcription 3, a member of the STAT protein family that plays critical roles in affecting cytokine-induced changes in gene expression. The *STAT3* variant in this patient is a biparentally verified *de novo* variant, but this variant was reported in 2011 (16) and has been included in the Human Gene Mutation Database (HGMD).

Up to date, about 224 STAT3 mutations have been identified with AD-HIES in HGMD. The majority of patients were with missense mutations located in the DNA-binding and SH2 domain of STAT3 protein; R382W, R382Q, and V637M are the most prevalent hotspot mutations, respectively.

In terms of clinical presentation, the most common infection sites are skin abscess (74.4%) and pneumonia (72%) (6). Similar to previous studies, an Indian cohort study showed that the majority of these STAT3-HIES patients presented with recurrent skin abscesses (77.7%) or pneumonia (62.9%) or both (59.2%); *mycobacterial* infections accounted for 18.5% (17). In comparison with USIDNET (6) and the French cohort study (18), Asian patients have the following characteristics: diagnosis at an older age, a higher risk of Bacillus Calmette-Guerin Vaccine complications, and a reduced rate of candidiasis and chronic otitis media (17, 19–21).

Owing to the pathophysiological mechanism of immunodeficiency causes, the infection has not been fully elucidated. The treatment of HIES remains difficult. No randomized clinical trials have been carried out due to the rarity of HIES. The current treatment is based on observational data and clinical experiences. Intensive care of dermatitis, immediate wide spectrum antibiotic, or antifungal treatment of infections and surgical drainage of abscesses are the fundamental management of HIES management. The role of hematopoietic cell transplantation (HSCT) in STAT3-HIES is emerging. It is evident that successful transplant beneficiaries have moved forward with the disease phenotype. However, the impact of HSCT on the disease's non-immunologic components is still unknown (8, 22). Recently, a recombinant humanized IgG monoclonal antibody, omalizumab, has been characterized to be closely associated with a reduction in serum IgE (23). It binds to free serum IgE and prevents the binding of IgE to high-affinity FcεRI receptor on the surface of mast cells and basophils (23, 24), reducing the activation of inflammatory cells (such as the degranulation of mast cells) and releasing various inflammatory mediators (25). The Food and Drug Administration (FDA) has licensed its use in adults and adolescents with moderate-to-severe persistent asthma or chronic idiopathic urticaria in 2003 and 2014, respectively. Several case reports and case series suggested that omalizumab could be used in allergic bronchopulmonary aspergillosis (26). In addition, its off-label use has been reported in several conditions in which IgE has an important pathogenic role, such as hyper-IgE syndrome. The present patient was prescribed omalizumab, firstly owing to her high serum IgE level, poor control of respiratory symptoms, and prolonged use of systemic corticosteroids. After an initial drop of the level, the serum IgE level remained stable. ABPA accompanied by asthma is often complicated by infections of the lower respiratory tract, especially in the immunocompromised patient, rendering treatment with corticosteroids difficult, and omalizumab should be considered in this situation. The patient's condition improved on therapy and did not experience recurrent infection yet. No clinically relevant abnormalities were observed.

We summarized six articles about omalizumab in the treatment of HIES (9–14) (Table 1). In these cases, most patients presented with atopic dermatitis and eczema as the main manifestation. After treatment with different doses of omalizumab, the skin lesion gradually became under control, along with the decrease of IgE levels in most patients. Pulmonary manifestation improvement was less focused on in previous articles. Only one case mentioned an improvement in ACT scores and FEV1 with omalizumab 375 mg every 2 weeks for 3 months in a 14-year-old African American male with AR-HIES. However, the alteration of the IgE levels was not mentioned (11). As omalizumab primarily reduces free IgE, it is not possible to distinguish between free IgE and IgE-omalizumab serum complexes using conventional measures of total serum IgE (27, 28). As one of the main manifestations of HIES, the level of serum IgE was followed regularly and remained stable recently after an initial fluctuation of the level. Although omalizumab treatment relieved the rash and respiratory symptoms in some cases, there is no clear uniformity in the dose of omalizumab given, and more research is needed to determine this. Wang considered that the use of omalizumab in atopic dermatitis documented an association between a serum IgE concentration of under 700 IU/ml and a more favorable clinical response (29). The use of total IgE levels to determine the efficacy of omalizumab is not entirely credible.

**Table 1:** Six articles related to omalizumab for HIES.

**References**	**Sex/age (y) (mutated gene)**	**Main symptoms**	**Medical history**	**Dose and period omazulimab**	**Serum IgE-level (IU/mL) (before/after)**	**Change in symptoms**
Bard et al. (10)	F/26 (-)	Eczematous dermatitis, asthma	Infectious bronchitis, pneumonias, cystitis	450 mg every 2 weeks, 3 m	1,083/–	Symptoms improved after 1 mo, complete remission after 3 mo
Marcotte (11)	M/14 (-)	Severe atopic dermatitis asthma, atopic keratoconjunctivitis	Frequent respiratory infections, skin abscesses, allergic rhinitis,	375 mg every 2 weeks, 3 m	9,408/–	No active eczematous, pruritus and photophobia, improved in both eyes, vision improved, ACT score and FEV1 increase
Chularojanamontri et al. (12)	F/32 (-)	Pruritic skin lesions	Scalp abscesses, alopecia	300 mg every 2 weeks, 1 m	17,300/–	Eczematous lesions improved
Akbaş et al. (13)	F/12 (-)	Flexural eczema, intense pruritus	Frequent upper respiratory tract infections, mentalmotor retardation	300 mg every 1 month, 5 m (no benefit) changed to 300 mg every 2 weeks, 1 m	3,800/1,376	Relief of the pruritus and skin lesions
Alonso-Bello et al. (9)	M/37 (-)	Atopic dermatitis, generalized itching, peeling	–	300 mg every 2 weeks, 6 w (effective) 350 mg every 2 weeks,4 y	12,700/439	After 6 weeks, eczematous lesions improved; after 4 years, skin lesions exacerbation
Gomes et al. (14)	M/33 (STAT3 mutation)	Recalcitranteczema,	Folliculitis, onychomycosis, respiratory infections, esophageal candidiasis	375 mg every 2 weeks, 12 m	11,802/8,660	skin lesions and pruritus improved

In addition to efficacy, the dose varied from case to case. In the first case of HIES treated with omalizumab reported by Susan Bard (10), the dosage was chosen mainly based on the maximum dose of omalizumab for atopic dermatitis. In subsequent cases, the reasons for the dose selection of omalizumab were not clearly expressed. As the role of IgE in HIES remains unknown, the optimal dose, the duration of therapy, and the predictive biomarkers of effectiveness for HIES with omalizumab need to be further explored. In addition to omalizumab, the use of dupilumab, a fully human monoclonal IgG4 antibody, may also improve atopic dermatitis and eosinophilic esophagitis with patients with STAT3-HIES (30). Also, prospective studies are required to confirm the effectiveness.

In conclusion, the diagnosis and treatment of HIES are challenging due to its rarity. A detailed medical history and the NIH scoring system can be used to help the diagnosis. Molecular genetic testing that are readily available on a clinical basis is the only reliable diagnostic approach. The treatment of omalizumab was demonstrated to be beneficial in cases of HIES with allergic abnormalities, for which therapeutic options are often limited. However, further studies and long-term follow-up are still required to provide sufficient evidence.

## Data Availability Statement

The original contributions presented in the study are included in the article/Supplementary Materials, further inquiries can be directed to the corresponding author/s.

## Ethics Statement

Written informed consent was obtained from the individual(s) for the publication of any potentially identifiable images or data included in this article.

## Author Contributions

YL collected data, performed literature search, wrote the first draft of the manuscript, searched the literature, helped with data interpretation, and participated in the drafting of the manuscript. YZ, MS, SC, HL, YC, RO, XS, and PY contributed to the discussion of results and to the review of the manuscript. All authors read and approved the manuscript.

## Funding

This work was supported by the National Natural Science Foundation of China (No. 81800043) and the Natural Science Foundation of Hunan (No. 2020JJ5818).

## Conflict of Interest

The authors declare that the research was conducted in the absence of any commercial or financial relationships that could be construed as a potential conflict of interest.

## Publisher's Note

All claims expressed in this article are solely those of the authors and do not necessarily represent those of their affiliated organizations, or those of the publisher, the editors and the reviewers. Any product that may be evaluated in this article, or claim that may be made by its manufacturer, is not guaranteed or endorsed by the publisher.

## Abbreviations

HIES: Hyper-IgE syndrome
AD: autosomal dominant heredity
AR: autosomal recessive heredity
WES: whole-exome sequencing
PID: primary immunodeficiency disorders
OMIM: Online Mendelian Inheritance in Man
CT: computed tomography
ABPA: allergic bronchopulmonary aspergillosis
NGS: next-generation sequence
BALF: bronchoalveolar lavage fluid
CMV: cytomegalovirus
EBV: Epstein-Barr virus
SMZ-TMP: sulfamethoxazole and trimethoprim
FEV1: forced expiratory volume in the first second
FVC: forced vital capacity
ACT: asthma control test
STAT3: signal transducer and activator of transcription 3
HGMD: Human Gene Mutation Database.
