# Supplementary figures and images for: Omalizumab for STAT3 Hyper-IgE Syndromes in Adulthood: A Case Report and Literature Review
Source: Front Med (Lausanne). 2022 May 4;9:835257. doi: 10.3389/fmed.2022.835257 (PMC9114644; doi:10.3389/fmed.2022.835257)

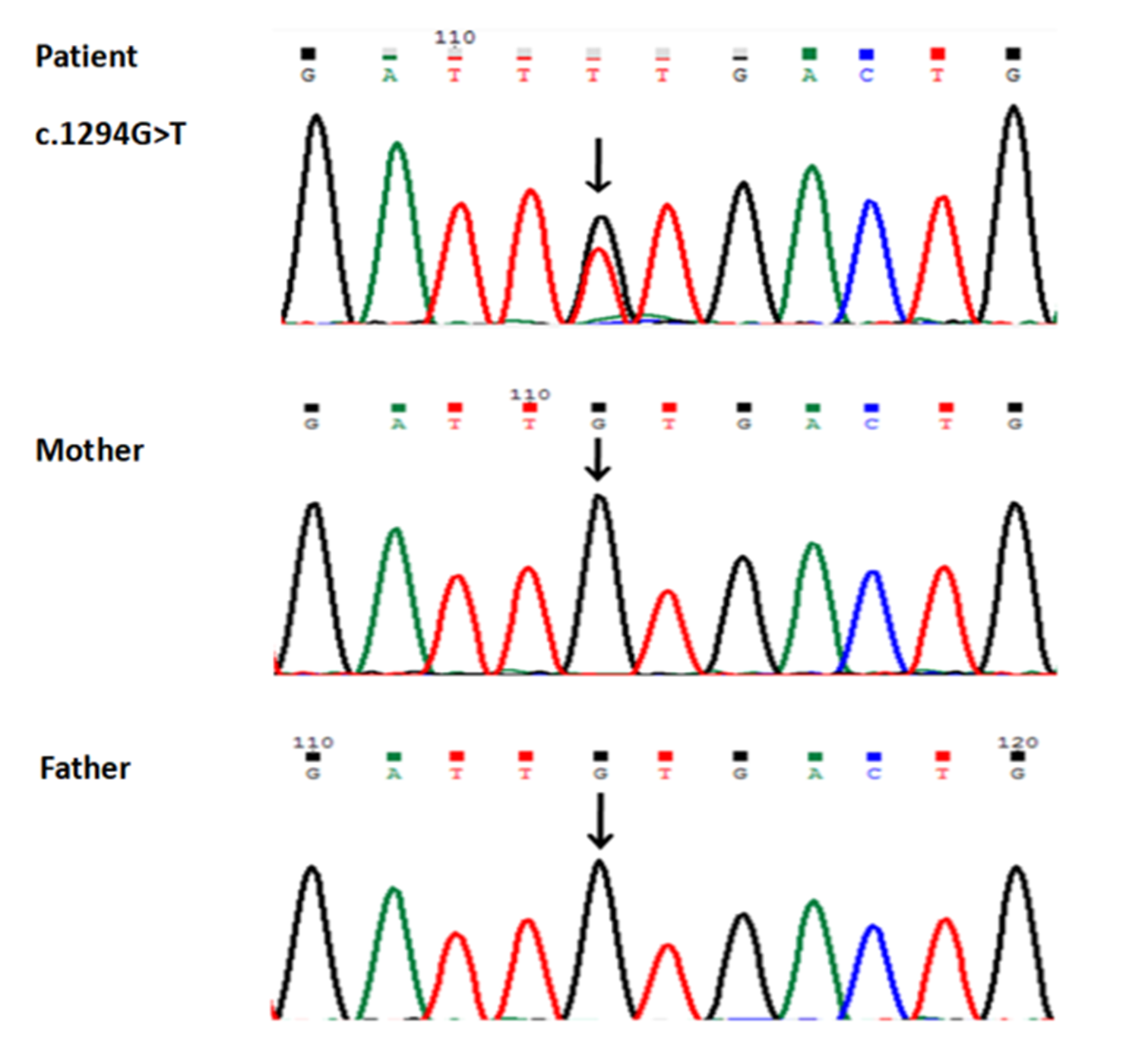

Supplement: Supplementary file 1 [file Image_1.png]

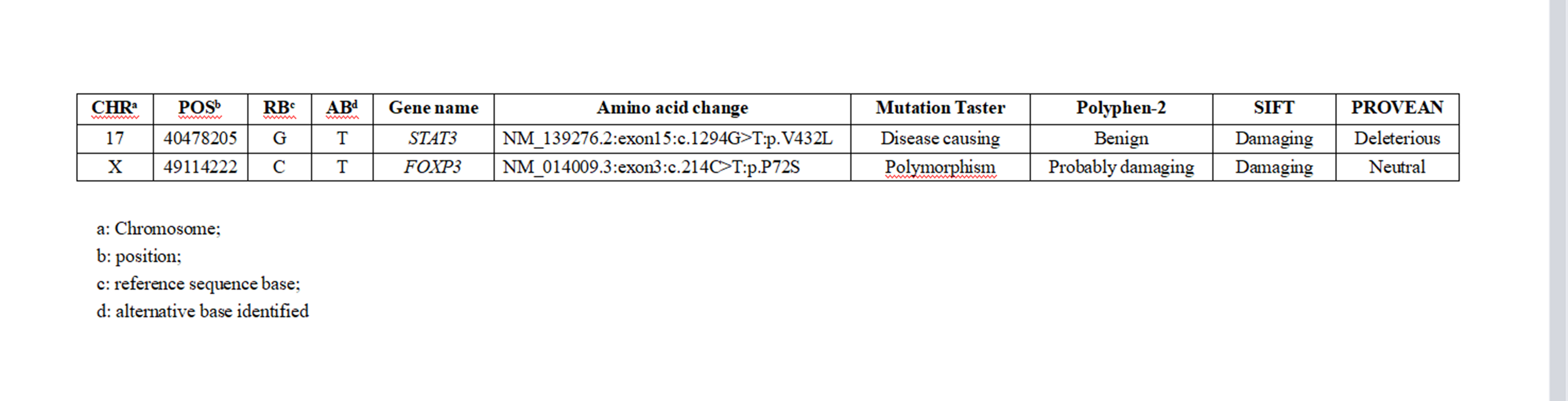

Supplement: Supplementary file 2 [file Image_2.png]

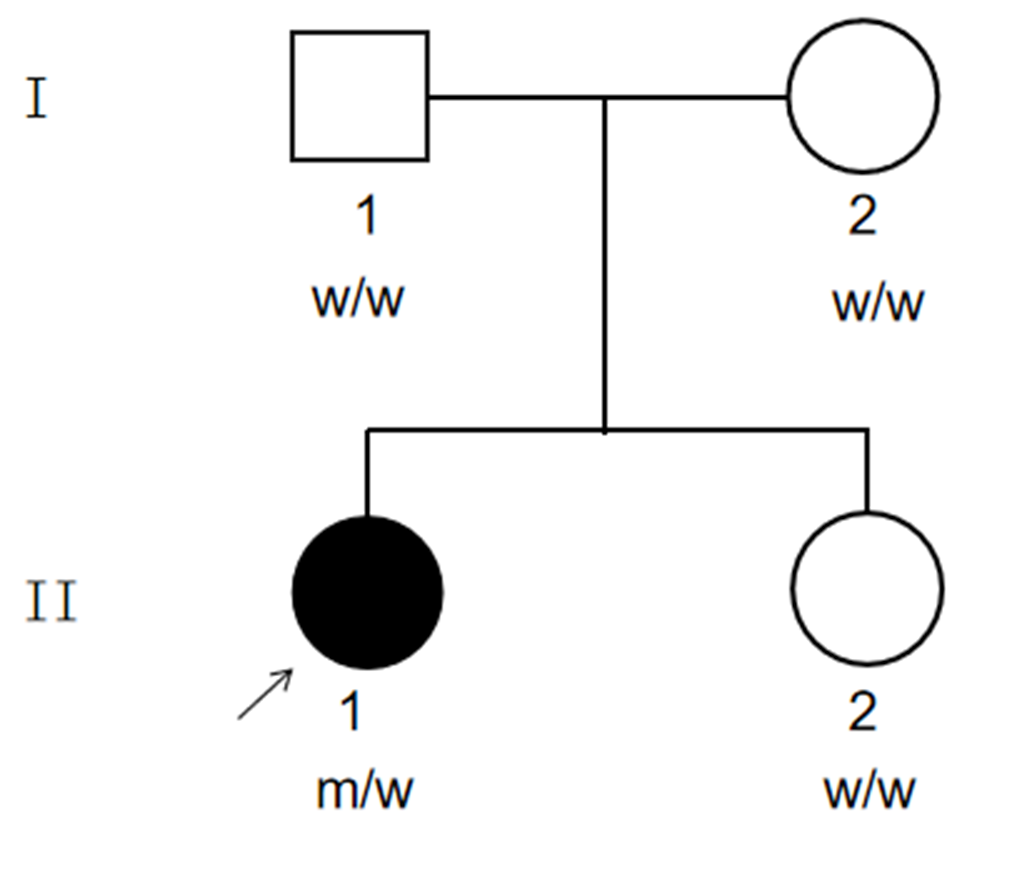

Supplement: Supplementary file 3 [file Image_3.png]
